# Prevalence of DSPN and Its Risk Factors Among Type 2 Diabetes Patients Attending Xuhang Community Health Service Center, in Shanghai, China

**DOI:** 10.1155/ije/5704920

**Published:** 2026-04-17

**Authors:** Fei Qi, Kan Ze, Jia Gao, Cheng Zhao, Yemin Cao

**Affiliations:** ^1^ Department of Vascular Medicine II, Shanghai TCM-Integrated Hospital, Shanghai University of Traditional Chinese Medicine, Shanghai, 200082, China, shutcm.edu.cn; ^2^ Department of Surgery VIII (Dermatology and Sores), Shanghai Municipal Hospital of Traditional Chinese Medicine, Shanghai University of Traditional Chinese Medicine, Shanghai, 200071, China, shutcm.edu.cn; ^3^ Department of Traditional Chinese Medicine, Xuhang Community Health Service Center, Shanghai, 201808, China; ^4^ Center of Vascular Medicine, Shanghai TCM-Integrated Hospital, Shanghai University of Traditional Chinese Medicine, Shanghai, 200082, China, shutcm.edu.cn

**Keywords:** diabetes, DPN, DSPN, screening

## Abstract

**Objective:**

This study aimed to investigate the prevalence of distal symmetrical polyneuropathy (DSPN) among Type 2 diabetes mellitus (T2DM) patients in the suburbs of Shanghai, China, and identify associated risk factors.

**Material and Methods:**

The screening process consisted of information registration, clinical consultation, and laboratory testing, which included hematological parameters, hepatic and renal function profiles, metabolic markers (glycemic control and lipid profiles), tumor biomarkers, and urinalysis. Additionally, the process involved screening tests for peripheral neuropathy, skin condition assessment, evaluation of toe deformities, and lower extremity vascular examination. The results were initially analyzed using univariate analysis, and variables with statistical significance were subsequently incorporated into logistic regression modeling to evaluate the associated risk factors.

**Results:**

The prevalence of DSPN was 45.53%. Univariate analysis found that diabetes duration, cardiovascular disease (CVD), dry skin, callus, dorsalis pedis artery pulsation (DPAP), posterior tibial artery pulsation (PTAP), popliteal artery pulsation (PP), age, red blood cell (RBC), low‐density lipoprotein (LDL), and creatinine (Cr) are statistically significant. The results of the logistic regression showed that age, dry skin, callus, and Cr are statistically significant among the risk factors for DSPN.

**Conclusion:**

T2DM patients in suburban areas exhibit a high prevalence of DSPN, with age, dry skin, callus, and elevated Cr levels serving as major risk factors for its development.

## 1. Introduction

As the most common chronic metabolic disease worldwide, the number of people suffering from diabetes is increasing dramatically. According to statistics, the global prevalence of diabetes was 9.3% in 2021, with approximately 537 million patients, and this number will rise to 783 million by 2045 if no active protective measures are taken [1].

Diabetic foot (DF) is one of the most serious complications of diabetes. DF is resource‐intensive to treat and requires interdisciplinary cooperation, with high treatment costs, long treatment cycles, low cure rates, and high recurrence rates [2]. In China, one survey showed that the total amputation rate due to diabetic foot ulcers (DFUs) was 19.03%, with major amputations accounting for 2.14% and minor amputations 16.88%. Patients with a previous history of foot ulcers have a 13‐fold higher risk of reoccurrence compared to those without such a history. Additionally, the risk of amputation (toe) is 2.0–10.5 times higher in these patients than in those without a history of foot ulcers [3]. Furthermore, the mortality rate among patients with amputations is 22% [4].

It is estimated that approximately half of all diabetes patients suffer from diabetic peripheral neuropathy (DPN) [5]. According to statistics, about a quarter of the medical expenses for diabetes treatment in the United States are spent on DPN [6]. DPN represents the most prevalent and one of the earliest complications of diabetes, affecting 33.5% of patients with Type 2 diabetes mellitus (T2DM) across multiple hospitals in Korea. This prevalence is notably higher than that of retinopathy (21.0%) and nephropathy (15.7%) [7]. A recent population‐based cross‐sectional study in China has shown that the prevalence of distal symmetrical polyneuropathy (DSPN) among patients with T2DM is as high as 67.6%, with the proportion of patients with painful neuropathy being 57.2%. Additionally, the prevalence of cardiac autonomic neuropathy (CAN) in patients with Type 1 diabetes (T1DM) and T2DM is 61.6% and 62.6%, respectively [8].

DPN represents a heterogeneous group of disorders that may involve various regions of the nervous system and exhibit a wide range of clinical manifestations. Among its diverse forms, DSPN is the most prevalent, comprising approximately 75% of all diabetic neuropathy cases [9, 10]. In China, the majority of DFUs are classified as the neuroischemic type [4]. During the progression of the disease, nerves and blood vessels exhibit complex interactions with each other. DSPN constitutes one of the key risk factors for the development of foot ulcers in individuals with diabetes [11], and in severe cases, it can potentially result in amputation [12].

Up to 50% of patients may be asymptomatic, which often leads to their condition being overlooked and subsequently delays in seeking medical care [13]. In the screening of DSPN, scales that incorporate clinical symptoms and signs, such as the Michigan diabetic neuropathy score (MDNS) [14], can be utilized. The neuropathy disability score (NDS) can also be used, which takes into account both screening and the severity of neuropathy [15]. For asymptomatic patients, assessment may involve 5 screening tests for peripheral neuropathy: pinprick sensation and thermal sensation, which reflect small‐fiber nerve function, as well as ankle reflex, vibration sense, and pressure sense, which indicate large‐fiber nerve function [16, 17]. During the consultation, if a comprehensive examination and detailed questioning of the medical history are not carried out, it is easy to be overlooked, resulting in a low detection rate of the disease. The relative lack of medical resources in Shanghai suburbs compared to urban areas and the lack of patients’ knowledge about diseases are also detrimental to the early detection of diseases [18].

Some experts and scholars in China have acknowledged the pivotal role of DSPN in the progression of DFUs. To facilitate early detection of DSPN, systematic studies focusing on its risk factors and pathogenesis mechanisms have been conducted. Screening study conducted by Yuan Xiaoyong in Beijing revealed that 47.1% of diabetic patients meeting the preliminary screening criteria exhibited foot lesions [19]. The results indicate that gender, duration of diabetes, presence of hyperlipidemia, chronic kidney disease, systolic blood pressure levels, low‐density lipoprotein (LDL), and high‐density lipoprotein (HDL) concentrations are significant risk factors associated with diabetic high‐risk feet. The study carried out by Li Jinlian at Southern Medical University demonstrates that the triglyceride and glucose index (TyG), glycosylated hemoglobin, Type A1C (HbA1c) levels, diabetes duration, presence of proteinuria, and age are significantly associated with the development of DSPN [20]. Lin Yueqing’s research demonstrates that the key risk factors for DSPN are the duration of diabetes, HbA1c levels, HDL, hemoglobin, and suboptimal treatment adherence [21]. Cao Du‐hua’s research demonstrates that age, diabetes duration, blood glucose levels, and the presence of comorbidities are significant risk factors associated with the development of DSPN [22]. An epidemiological study carried out in Taiwan in 2003 identified key characteristics of patients with DF: older age, a higher male‐to‐female ratio, longer diabetes duration, a history of smoking, suboptimal glycemic control, a greater proportion of insulin users, the presence of hypertension and hyperlipidemia, lower educational levels, and a tendency to reside in rural areas [23].

In this study, we hope to find the risk factors associated with the occurrence of DSPN by conducting screening for this disease in community hospitals, and to improve the sensitivity of screening and the detection rate of the disease in the future.

## 2. Methods and Materials

### 2.1. Study Design

We performed a cross‐sectional study of patients with diabetes attending this single institution. We have followed the Strengthening the Reporting of Observational Studies in Epidemiology (STROBE) guidelines for the reporting of this study.

### 2.2. Study Area and Duration

All patients included in this study are long‐term residents of Xuhang Town, Jiading District, Shanghai. The Jiading District Xuhang Town Community Health Service Center, serving as the local primary care facility, is responsible for the ongoing follow‐up and routine health examinations of this population. The screening period was 2023.8.2–2023.8.19. Individuals who participated in diabetes follow‐up programs and regular check‐ups at this community‐based primary care center and met the established diagnostic criteria were enrolled in the study. Patients with ulcers and unhealed wounds are excluded.

This study was approved by the Ethics Committee of Shanghai Traditional Chinese Medicine (TCM)‐Integrated Hospital (20200971).

### 2.3. Detection Methods and Instruments

In collaboration with the Community Health Service Center of Xuhang Town, Jiading District, an 18‐day health screening campaign was conducted. Patients registered with family doctors were systematically organized into groups based on their respective villages for staged participation in the screening process. Between 50 and 110 individuals were screened each day. The screening venue was fully equipped with a biochemical testing room, a neurological lesion detection tool kit (10‐g Semmes–Weinstein monofilament, 128‐Hz tuning fork, Tip Therm, pins, and a percussion hammer), and professional medical personnel. The screening procedure encompassed information registration, clinical consultation, biochemical blood and urinal analysis, neuropathy examination, skin condition assessment, evaluation of toe deformities, vascular palpation, and other essential steps. Each patient’s examination duration was approximately 15–20 min. All medical histories were provided orally by the patients, and biochemical blood analysis was done by the laboratory department according to hospital routines. Neuropathy screening examinations and vascular examinations are performed by professionals with relevant training. Detailed information regarding the specific operational methods can be found in the Supporting Information.

### 2.4. Diagnostic Criteria

Since this study was conducted in a community hospital located in a suburban area of Shanghai, China, the diagnostic criteria for both T2DM and DSPN refer to the Chinese guidelines [16]:

T2DM: Typical diabetes symptoms with random blood sugar ≥ 11.1 mmol/L, or fasting blood sugar ≥ 7.0 mmol/L; or oral glucose tolerance test (OGTT) ≥ 11.1 mmol/L; or HbA1c ≥ 6.5%.

DSPN: (1) Clear history of diabetes; neuropathy occurring at or after diagnosis of diabetes; (2) 5 screening tests for peripheral neuropathy: (1) pressure sensation, (2) vibration sensation, (3) pinprick sensation, (4) thermal sensation, (5) ankle reflex. (For the specific procedures, see Section 2.1 in Supporting Information 2.)

The first criterion must be satisfied. Two of the 5 screening tests are abnormal and can be clinically diagnosed as DSPN.

### 2.5. Independent Variables

The analysis included the sociodemographic and clinical characteristics of the affected population, including duration of disease, smoking, and clinical status, such as hypoglycemic regimen and skin condition (dry skin, furfur, chap, corns, callus, and toe abnormalities; for detailed information, refer to Supporting Information 2, Section 2.3.). Lower extremity vascular status was assessed by dorsalis pedis artery pulsation [DPAP], posterior tibial artery pulsation [PTAP], popliteal artery pulsation [PP] (for detailed information, refer to Supporting Information 2, Section 2.2.). Anthropometric measurements included waist and hip measurements, body mass index (BMI), and waist‐to‐hip ratio. Laboratory examination comprised hematological parameters, hepatic and renal function profiles, metabolic markers (glycemic control and lipid profiles), tumor biomarkers, and urinalysis.

### 2.6. Statistical Methods

This study used R language (Version 4.3.1) and R Studio (2023.06.1 + 524) for statistical analysis. Discrete data were expressed as number (percentage), and continuous data were expressed as mean (standard deviation). First, a one‐way analysis was performed, and then, positive indicators were selected for logistic regression analysis. The preset α = 0.05. For more details, refer to Supporting Information 3.

## 3. Results

A total of 850 patients with T2DM were included in the study. Detailed data are provided in the Supporting Information and Table 1. Among these 850 patients, the mean fasting blood glucose (FBG) was 6.83 mmol/L, with a median of 6.3 mmol/L; the mean HbA1c was 7.00%, with a median of 6.7%. These research results suggest that blood sugar control among patients participating in the screening was suboptimal.

**FIGURE 1:**
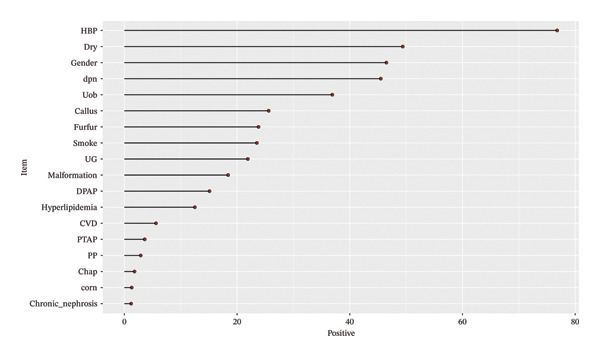
This dot plot shows the rate of positivity for discrete variables, as seen in the highest rates of hypertension, dryness, and urinary occult blood.

The prevalence of DSPN was 45.53%. Figure 1 presents a dot plot illustrating the positivity rates for discrete variables, with the highest rates observed for hypertension, dryness, and UOB. Based on the data and statistical results in Tables 1, 2, 3, the following variables were found to be statistically significant in the univariate analysis: diabetes duration, cardiovascular disease (CVD), dryness, callus, DPAP, PTAP, PP, age, red blood cell [RBC], LDL, and creatinine (Cr). Consequently, these indicators were selected for logistic regression analysis.

**TABLE 1:** Demographic and clinical characteristics.

Items	Level	Overall (*N* = 850)	No DSPN (*N* = 463)	DSPN (*N* = 387)	*p*
Gender (%, *same below*)	Male	455 (53.5)	243 (52.5)	212 (54.8)	0.549
	Female	395 (46.5)‐	220 (47.5)	175 (45.2)	

Diabetes duration	≤ 8 years	399 (46.9)	220 (47.5)	179 (46.3)	**0.05**
	8 ∼ 15	318 (37.4)	183 (39.5)	135 (34.9)	
	> 15	133 (15.6)	60 (13.0)	73 (18.9)	

Smoke	No	650 (76.5)	356 (76.9)	294 (76.0)	0.815
	Yes	200 (23.5)	107 (23.1)	93 (24.0)	

Hypertension	No	197 (23.2)	110 (23.8)	87 (22.5)	0.72
	Yes	653 (76.8)	353 (76.2)	300 (77.5)	

Hyperlipidemia	No	744 (87.5)	415 (89.6)	329 (85.0)	0.054
	Yes	106 (12.5)	48 (10.4)	58 (15.0)	

Chronic nephrosis	No	840 (98.8)	461 (99.6)	379 (97.9)	0.06
	Yes	10 (1.2)	2 (0.4)	8 (2.1)	

CVD	No	802 (94.4)	444 (95.9)	358 (92.5)	**0.047**
	Yes	48 (5.6)	19 (4.1)	29 (7.5)	

Age (mean(SD), *same below*)		70.30 (6.30)	69.26 (6.11)	71.53 (6.30)	**<** **0.001**
Weight		61.96 (10.31)	62.24 (10.48)	61.62 (10.11)	0.379
Height		160.10 (8.19)	160.17 (7.98)	160.03 (8.43)	0.802
BMI		24.12 (3.22)	24.22 (3.36)	23.99 (3.04)	0.3
Waistline		89.32 (8.35)	89.26 (8.74)	89.39 (7.86)	0.821
Hipline		97.24 (6.60)	97.24 (6.83)	97.24 (6.31)	0.999
WHR		0.92 (0.05)	0.92 (0.05)	0.92 (0.05)	0.536

*Note:* Bold values indicate *p* < 0.05, indicating a statistically significant difference.

**TABLE 2:** Skin conditions, toe abnormalities, and lower extremity arterial pulsation status.

Items	Level	Overall (*N* = 850)	No DSPN (*N* = 463)	DSPN (*N* = 387)	*p*
Dry skin	No	430 (50.6)	278 (60.0)	152 (39.3)	**<** **0.001**
	Yes	420 (49.4)	185 (40.0)	235 (60.7)	

Furfur	No	648 (76.2)	365 (78.8)	283 (73.1)	0.062
	Yes	202 (23.8)	98 (21.2)	104 (26.9)	

Chap	No	835 (98.2)	455 (98.3)	380 (98.2)	0.999
	Yes	15 (1.8)	8 (1.7)	7 (1.8)	

Corn	No	839 (98.7)	455 (98.3)	384 (99.2)	0.358
	Yes	11 (1.3)	8 (1.7)	3 (0.8)	

Callus	No	632 (74.4)	364 (78.6)	268 (69.3)	**0.002**
	Yes	218 (25.6)	99 (21.4)	119 (30.7)	

Malformation	No	694 (81.6)	382 (82.5)	312 (80.6)	0.536
	Yes	156 (18.4)	81 (17.5)	75 (19.4)	

DPAP	No	722 (84.9)	409 (88.3)	313 (80.9)	**0.003**
	Yes	128 (15.1)	54 (11.7)	74 (19.1)	

PTAP	No	819 (96.4)	456 (98.5)	363 (93.8)	**0.001**
	Yes	31 (3.6)	7 (1.5)	24 (6.2)	

PP	No	825 (97.1)	457 (98.7)	368 (95.1)	**0.004**
	Yes	25 (2.9)	6 (1.3)	19 (4.9)	

*Note:* Bold values indicate *p* < 0.05, indicating a statistically significant difference.

**TABLE 3:** Laboratory examinations.

Items	Level	Overall (*N* = 850)	No DSPN (*N* = 463)	DSPN (*N* = 387)	*p*
RBC		4.42 (0.46)	4.46 (0.47)	4.38 (0.45)	**0.023**
Hb		137.30 (14.49)	137.96 (14.89)	136.50 (13.98)	0.143
HCT		40.92 (4.03)	41.15 (4.04)	40.64 (4.00)	0.065
MCV		92.71 (4.60)	92.56 (4.35)	92.89 (4.88)	0.296
MCH		31.12 (1.70)	31.08 (1.67)	31.18 (1.74)	0.386
MCHC		335.83 (10.05)	335.89 (9.60)	335.77 (10.58)	0.871
WBC		6.11 (1.47)	6.09 (1.47)	6.13 (1.47)	0.701
N		60.84 (8.20)	60.79 (7.88)	60.90 (8.57)	0.844
L		29.83 (7.56)	30.00 (7.35)	29.62 (7.80)	0.462
PLT		192.03 (59.70)	191.55 (58.58)	192.60 (61.08)	0.798
AST		22.46 (10.34)	22.30 (10.03)	22.65 (10.72)	0.632
ALT		22.72 (15.87)	22.87 (15.89)	22.54 (15.86)	0.761
Tbil		15.68 (6.30)	16.07 (6.19)	15.23 (6.40)	0.052
TC		4.79 (1.25)	4.84 (1.21)	4.73 (1.29)	0.175
LDL		2.77 (0.90)	2.83 (0.91)	2.70 (0.90)	**0.038**
HDL		1.26 (0.55)	1.28 (0.69)	1.23 (0.31)	0.218
TG		1.91 (1.42)	1.91 (1.44)	1.90 (1.40)	0.965
BUN		6.41 (1.95)	6.30 (1.85)	6.55 (2.06)	0.064
Cr		80.70 (32.19)	76.13 (23.14)	86.16 (39.80)	**< 0.001**
UA		340.60 (88.85)	338.03 (89.10)	343.67 (88.57)	0.356
HbAlc		7.00 (1.29)	7.00 (1.30)	7.00 (1.28)	0.979
FBG		6.83 (2.31)	6.76 (2.20)	6.91 (2.43)	0.358
CEA		4.30 (9.36)	4.37 (11.95)	4.21 (4.66)	0.8
CA199		11.13 (31.36)	11.97 (41.21)	10.14 (11.37)	0.397
SG		1.03 (0.19)	1.03 (0.18)	1.03 (0.20)	0.95
UMA		68.99 (74.22)	65.26 (72.80)	73.44 (75.73)	0.109

UOB	No	536 (63.1)	290 (62.6)	246 (63.6)	0.971
	+	239 (28.1)	132 (28.5)	107 (27.6)	
	++	58 (6.8)	31 (6.7)	27 (7.0)	
	+++	17 (2.0)	10 (2.2)	7 (1.8)	

UG	No	664 (78.1)	362 (78.2)	302 (78.0)	0.504
	+	54 (6.4)	30 (6.5)	24 (6.2)	
	++	31 (3.6)	13 (2.8)	18 (4.7)	
	+++	101 (11.9)	58 (12.5)	43 (11.1)	

*Note:* PP: popliteal artery pulsation; Hb: hemoglobin; HCT: hematocrit; N: neutrophil; L: lymphocyte; Plt: platelets; AST: aspartate aminotransferase; ALT: alanine aminotransferase; TG: triglyceride; Cr: creatinine; HbAlc: glycosylated hemoglobin, Type A1C; UMA: urinary microalbumin; UG: glucose in urine. Bold values indicated a *p* value less than 0.05, indicating a statistically significant difference.

Abbreviations: BMI, body mass index; BUN, blood urea nitrogen; CA199, carbohydrate antigen 199; CEA, carcinoembryonic antigen; CVD, cardiovascular disease; DPAP, dorsalis pedis artery pulsation; FBG, fasting blood glucose; HDL, high‐density lipoprotein; LDL, low‐density lipoprotein; MCH, mean corpuscular hemoglobin; MCHC, mean corpuscular hemoglobin concentration; MCV, mean corpuscular volume; PTAP, posterior tibial artery pulsation; RBC, red blood cell; SG, specific gravity; Tbil, total bilirubin; TC, total cholesterol; UA, uric acid; UOB, urine occult blood; WBC, white blood cell; WHR, waist‐to‐hip ratio.

**FIGURE 2:**
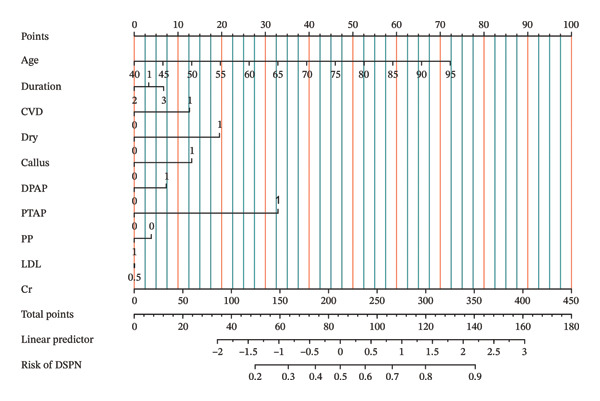
This nomogram of the logistic regression model risk factors showed that age and Cr play an important role in the development of DSPN.

**FIGURE 3:**
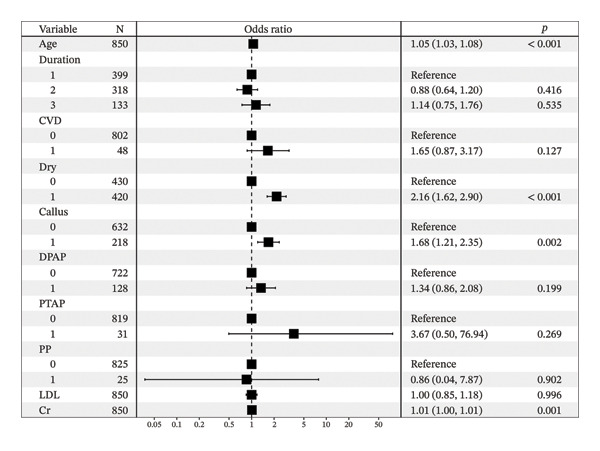
This forest plot shows the odds ratio and its 95% CI for each risk factor for the logistic regression model.

The results of the logistic regression of Table 4 showed that age, dry skin, callus, and Cr are statistically significant among the risk factors for DSPN. The Nomogram (Figure 2) of the logistic regression model risk factors showed that age and Cr play an important role in the development of DSPN. Forest plot (Figure 3) showed the odds ratio and its 95% CI for each risk factor for the logistic regression model.

**TABLE 4:** Logistic regression results for DSPN risk factors.

Term	Estimate	std.error	Statistic	exp(coef) [confint]	*p*
Age	0.05	0.01	4.21	1.05 [1.03, 1.08]	**< 0.001**
Diabetes duration	−0.13	0.16	−0.81	0.88 [0.64, 1.20]	0.42
CVD	0.50	0.33	1.53	1.65 [0.87, 3.17]	0.13
Dry skin	0.77	0.15	5.17	2.16 [1.62, 2.90]	**< 0.001**
Callus	0.52	0.17	3.07	1.68 [1.21, 2.35]	**< 0.001**
DPAP	0.29	0.23	1.28	1.34 [0.86, 2.08]	0.20
PTAP	1.30	1.18	1.11	3.67 [0.50, 76.94]	0.27
PP	−0.15	1.26	−0.12	0.86 [0.04, 7.87]	0.90
LDL	0.00	0.08	0.00	1.00 [0.85, 1.18]	1.00
Cr	0.01	0.00	3.19	1.01 [1.00, 1.01]	**0.001**

*Note:* Bold values indicate *p* < 0.05, indicating a statistically significant difference.

## 4. Discussion

According to this study, the prevalence of DSPN among T2DM patients who participated in the screening was 45.53%. A screening study conducted by Yuan Xiaoyong in Beijing revealed that 47.1% of diabetic patients meeting the preliminary screening criteria exhibited foot lesions, comparable to the findings of this screening study [19]. Additionally, research carried out by Zhou Guiyang in Jinshan, Shanghai, indicated a DF risk ratio of 23.6% [24], significantly lower than the findings of this study. This study emphasized the critical importance of vascular examination. They integrated Doppler ultrasound examination into the screening program, thereby refining the screening criteria and improving the accuracy of the results.

DPSN occurs in at least 10%–15% of newly diagnosed persons with T2DM, with rates increasing to 50% after 10 years of disease duration. It may even be present in up to 10%–15% of people with impaired glucose tolerance (prediabetes) [25]. According to the preliminary literature review conducted during the initial phase of the study [26, 27], the disease course was categorized using 8 years and 15 years as the predefined cutoff points. We found in this study that the older the age, the higher the prevalence of DSPN (OR 1.05, 1.03–1.08, 95% CI). In univariate comparative analysis, disease duration was statistically significant. However, it was not statistically significant in logistic regression. Based on the findings of other scholars, age and disease duration may represent the primary risk factors for DSPN [19–22]. Additionally, in this community‐dwelling population screening, the majority were elderly individuals with a mean age of 70 years and a median age of 70 years (see Table 1 in Supporting Information 1). Consequently, the influence of disease duration on the study outcomes may be subject to some degree of bias. Moreover, survival bias and variations in diagnostic methods could also impact the role of disease duration. For instance, patients with longer disease duration might experience mortality due to severe complications (e.g., cardiovascular events), leading to an underrepresentation of long‐duration DSPN cases in the study sample.

The findings of this screening demonstrated that the development of DSPN was significantly associated with dry skin and corn formation. The development of dryness is closely associated with long‐term hyperglycemia and autonomic neuropathy in patients with diabetes. Chronic hyperglycemia promotes the accumulation of advanced glycation end products (AGEs) in the dermis, leading to reduced sweat secretion and decreased hydration of the stratum corneum, which in turn causes dryness, cracking, and other forms of damage to the skin on the feet [28]. And foot care is very important for people with diabetes [29]. When the skin barrier of the foot is abnormal, such as dryness and desquamation, it will increase the risk of skin collapse and even foot infection. Callus refers to the localized thickening and hardening of the foot’s keratinized layer, which occurs as a result of excessive mechanical pressure. In severe cases, it may compromise balance function and lead to abnormal gait [30]. During ambulation, local pressure becomes abnormally elevated [31], and DSPN further exacerbates the development of callus. Diabetic motor neuropathy can induce foot muscle damage, subsequently causing structural alterations in the foot and deformities, such as hammer toes, mallet toes, or claw toes.

The pathogenesis of DSPN has not been fully elucidated, and some experiments have confirmed that the occurrence of DSPN is related to microangiopathy [32, 33]. Diabetic microcirculatory dysfunction represents a specific pathological alteration in diabetes. Chronic hyperglycemia facilitates the formation of AGEs, which subsequently impair vascular endothelial cells and nerve cells, thereby contributing to microvascular complications [34]. Microvascular lesions can induce cellular ischemia and hypoxia, subsequently leading to direct impairment of nerve cell function and ultimately contributing to DSPN [35]. The main lesions of DSPN are capillary basement membrane thickening, endothelial cell degeneration, swelling, hyperplasia, and glycoprotein accumulation to increase the thickness of the vascular wall, which leads to narrowing of the vascular lumen and increase in resistance, and it can further increase vascular sclerosis in the nervous system, reduce its contraction–diastolic function, and lead to blood oxygen irrigation in nerve tissues [33]. RBC and Cr may both be correlated with microcirculatory disorders. Some scholars have found in their studies that early indicators of renal impairment. Zou et al. found that compared with patients with diabetic nephropathy alone, patients with concomitant DSPN had a more pronounced degree of renal dysfunction, lower serum C‐peptide levels, and concomitant peripheral nerve conduction dysfunction [36]. Pu Chengkun et al. concluded that early kidney injury indicators have a certain predictive and analytical value for DSPN and found that N‐acetyl‐β‐D‐glucosaminidase/Cr (NAG/Cr) has a high value for clinical prediction of DSPN. It is consistent with the results of this study. Foreign studies have also found that for every 10 μmol/L increase in Cr, the OR value rises by 1.07 [37].

Patients with T2DM often exhibit dyslipidemia, which may increase their susceptibility to vascular complications [38]. This condition is characterized by reduced levels of HDL and elevated levels of TC, TG, and LDL. These changes result in neurotrophic dysfunction, leading to neuronal ischemia and hypoxia, as well as damage to the structure of nerve fibers. This constitutes a key pathological basis for DSPN associated with lipid metabolic disorders [39]. HDL serves as a protective factor by facilitating the removal of harmful substances, such as TC, TG, and LDL, which are deposited in the vascular intima, thereby exerting an antiatherosclerotic effect.

However, the development of atherosclerotic plaques may cause narrowing of the lumen in neurotrophic blood vessels, leading to impaired microcirculation, ischemia, and hypoxia in the peripheral nervous system, and ultimately resulting in nerve cell injury [40].

As a common complication of diabetes, vascular lesions not only affect large and medium arteries but also markedly impact small arteries below the knee. The primary pathological features include medial calcification, segmental narrowing, or occlusion of vessels, which are among the key factors contributing to DF syndrome [4]. Studies have demonstrated that the development of DPN is closely associated with hemodynamic alterations in lower extremity arteries. Qiang Zhou et al., utilizing color Doppler ultrasound technology, observed that in patients with mild DPN, no significant hemodynamic changes were detected in the large arteries of the lower extremities, whereas an increase in blood flow velocity and volume was noted in the distal small vessels [41]. Some studies have investigated the association between carotid intima–media thickness (CIMT) and DPN. There is an interaction between blood vessels and nerves, nerves can promote angiogenesis, and blood vessels can also provide nutrients needed for nerves [40, 42]. The formation of atherosclerotic plaque can cause the narrowing of neurotrophic vascular lumen, interfere with microcirculation, and lead to peripheral nervous system ischemia, hypoxia, and eventually damage nerve cells. The sequence of occurrence of vascular disease and peripheral neuropathy is not clear in the current study, but there is an indisputable fact that there is mutual influence between them.

Many studies have found that the level of blood sugar control is closely related to the occurrence of DSPN [20–22, 38, 39]. Our research findings indicate that the mean FBG level was 6.83 mmol/L, with a median of 6.3 mmol/L; the mean HbA1c level was 7.00%, with a median of 6.7% (see Supporting Information 1 Table 1). The level of blood sugar control is closely associated with the development of microcirculatory disorders [16]. Studies have demonstrated that in elderly patients with T2DM, the risk of developing DPN increases significantly as the HbA1c level reaches or exceeds 8.49% [43].

The aim of this research is to provide a theoretical foundation for the early detection of DSPN. Given China’s large population, there is a pronounced disparity in the allocation of medical resources between urban and rural areas, as well as in periurban regions. This results in limited disease awareness and suboptimal treatment adherence among patients in suburban areas, thereby complicating early disease detection. Stratified screening for high‐risk populations can significantly enhance screening efficiency while minimizing resource waste. However, current studies exhibit considerable variability in indicator selection, screening methodologies, screening locations, and target populations, precluding the establishment of a systematic and standardized screening protocol. Moving forward, the integration of artificial intelligence technologies and multiomics analytical approaches should be leveraged to refine screening processes. Additionally, efforts must focus on strengthening the screening capabilities of primary healthcare institutions, thereby establishing a more precise and efficient classification‐based screening and management system for DSPN.

This study was only conducted in Xuhang Town, Jiading District, Shanghai, and the data have certain limitations. The screening scope can be gradually expanded in the future. At present, this study was limited to data collected over a single year (2023), and for this part of patients, further follow‐up can be conducted in the future. The conclusions drawn at this stage of the study need further research to support.

**Nomenclature**
AST: Aspartate aminotransferase
ALT: Alanine aminotransferase
AGEs: Advanced glycation end products
BMI: Body mass index
BUN: Blood urea nitrogen
CA199: Carbohydrate antigen 199
CAN: Cardiac autonomic neuropathy
CEA: Carcinoembryonic antigen
CIMT: Carotid intima–media thickness
Cr: Creatinine
CVD: Cardiovascular disease
DF: Diabetic foot
DPN: Diabetic peripheral neuropathy
DFUs: Diabetic foot ulcers
DPAP: Dorsalis pedis artery pulsation
DSPN: Distal symmetrical polyneuropathy
FBG: Fasting blood glucose
HB: Hemoglobin
HbA1c: Glycosylated hemoglobin, Type A1C
HCT: Hematocrit
HDL: High‐density lipoprotein
L: Lymphocyte
LDL: Low‐density lipoprotein
MCH: Mean corpuscular hemoglobin
MCHC: Mean corpuscular hemoglobin concentration
MCV: Mean corpuscular volume
MDNS: Michigan diabetic neuropathy score
N: Neutrophil
NAG: N‐Acetyl‐β‐D‐glucosaminidase
NDS: Neuropathy disability score
OGTT: Oral glucose tolerance test
Plt: Platelets
PP: Popliteal artery pulsation
PTAP: Posterior tibial artery pulsation
RBC: Red blood Cell
SG: Specific gravity
STROBE: Strengthening the Reporting of Observational Studies in Epidemiology
Tbil: Total bilirubin
TC: Total cholesterol
TCM: Traditional Chinese Medicine
T2DM: Type 2 diabetes mellitus
TG: Triglyceride
TyG: Triglyceride–glucose index
UA: Uric acid
UG: Glucose in urine
UMA: Urinary microalbumin
UOB: Urine occult blood
WBC: Wight blood cell
WHR: Waist‐to‐hip ratio

## Author Contributions

Fei Qi and Kan Ze have contributed equally to this work.

Fei Qi: data organization and DSPN screening; Kan Ze: data analysis; Jia Gao and Cheng Zhao: recording patient information and routine examination; and Yemin Cao: design and supervision.

## Funding

This work was supported by Health Commission of Traditional Chinese Medicine Research Project, Hongkou District, Shanghai (HKQGYQY‐ZYY‐2022‐14); the National Natural Science Foundation of China (82174382); the Construction Project of the National Primary Inheritance Studio for Famous Senior Chinese Medicine Expert Cao Yemin (Z155080000004); and the 13th Five‐Year Clinical Key Specialties of Shanghai Municipal Healthcare Commission (Surgery of Traditional Chinese Medicine).

## Consent

The authors have nothing to report.

## Conflicts of Interest

The authors declare no conflicts of interest.

## Supporting Information

Additional supporting information can be found online in the Supporting Information section. Supporting Information

## Supporting information


**Supporting Information 1** Supporting Information 1 contains detailed statistical results for continuous variables.

**Supporting Information 2** Supporting Information 2 contains screening tests for peripheral neuropathy, lower extremity vascular examination, and skin condition and toe abnormalities.

**Supporting Information 3** Supporting Information 3 includes the R packages and commands used for statistical analysis and graphing.

## Data Availability

The datasets supporting the conclusions of this article are included within the article or supporting information.
