# Supplementary material for: Prevalence of DSPN and Its Risk Factors Among Type 2 Diabetes Patients Attending Xuhang Community Health Service Center, in Shanghai, China
Source: Int J Endocrinol. 2026 Apr 17;2026:5704920. doi: 10.1155/ije/5704920 (PMC13088976; doi:10.1155/ije/5704920)
Supplement: Supplementary file 1 — Supporting Information 1 Supporting Information 1 contains detailed statistical results for continuous variables. [file IJE-2026-5704920-s002.pdf]

Supplementary material 1

Descriptive Statistic(Continuous variable)

var	min	max	median	q1	q3	iqr	mad	mean	sd	se	ci
age	42	94	70	66	74	8	5.93	70.296	6.298	0.216	0.424
BMI	15.24	38.39	23.875	21.932	26.04	4.108	3.099	24.118	3.216	0.11	0.217
BUN	2.5	21.2	6.1	5.1	7.3	2.2	1.631	6.413	1.952	0.067	0.131
CA199	2	794.4	6.61	3.832	11.87	8.038	5.145	11.133	31.362	1.076	2.111
CEA	0.58	243	3.245	2.22	4.628	2.408	1.698	4.297	9.361	0.321	0.63
Cr	41.06	437.16	73.92	62.502	88.745	26.243	18.592	80.7	32.192	1.104	2.167
FBG	2.6	20.6	6.3	5.4	7.575	2.175	1.483	6.829	2.31	0.079	0.156
GOT	8	114	20	17	24	7	5.93	22.46	10.344	0.355	0.696
GPT	5	159	18	14	25	11	7.413	22.719	15.871	0.544	1.068
HbAlc	4.8	12.7	6.7	6.1	7.6	1.5	1.038	7.002	1.29	0.044	0.087
HCT	23	55.4	40.95	38.4	43.5	5.1	3.781	40.92	4.03	0.138	0.271
HDL	0.59	13.2	1.185	1.02	1.397	0.377	0.274	1.257	0.553	0.019	0.037
height	122	184.5	160	154	166	12	8.896	160.103	8.185	0.281	0.551
hipline	63	119	97	93	101	8	5.93	97.235	6.595	0.226	0.444
hp	32.5	185	138	128	147	19	13.343	137.297	14.49	0.497	0.976
L	1.38	55.5	29.6	24.8	34.1	9.3	6.968	29.83	7.556	0.259	0.509
LDL	0.74	6.18	2.755	2.122	3.4	1.277	0.941	2.769	0.903	0.031	0.061
MCH	23.6	39	31.1	30.1	32.1	2	1.483	31.123	1.702	0.058	0.115
MCHC	303	372	336	329.25	342	12.75	8.896	335.834	10.054	0.345	0.677

MCV	74	120	93	90	95	5	4.448	92.706	4.598	0.158			0.31
N	score	850	2.38	84	61.3	56.1	66.3	10.2	7.561	60.84	8.196	0.281	0.552
plt	score	850	7	404	189	151.25	226	74.75	55.597	192.032	59.696	2.048	4.019
RBC	score	850	2.08	6.02	4.425	4.16	4.72	0.56	0.423	4.423	0.461	0.016	0.031
SG	score	850	1	5	1.025	1.02	1.025	0.005	0.007	1.032	0.193	0.007	0.013
TBil	score	850	4.3	62.8	14.4	11.4	18.8	7.4	5.189	15.684	6.298	0.216	0.424
TC	score	850	1.83	11.11	4.67	3.94	5.54	1.6	1.216	4.789	1.246	0.043	0.084
TG	score	850	0.34	15.89	1.56	1.09	2.19	1.1	0.771	1.905	1.422	0.049	0.096
UA	score	850	129	660	334.5	275	398	123	91.18	340.598	88.85	3.048	5.982
UMA	score	850	5	250	36	22	78	56	28.911	68.987	74.219	2.546	4.997
waistline	score	850	62	120	89	84	95	11	8.896	89.316	8.349	0.286	0.562
WBC	score	850	2.6	12	5.9	5.025	7	1.975	1.334	6.106	1.471	0.05	0.099
weight	score	850	36.5	102	61.5	55	68.975	13.975	9.637	61.958	10.314	0.354	0.694
WHR	score	850	0.7	1.06	0.92	0.883	0.95	0.067	0.044	0.918	0.05	0.002	0.003
