# Supplementary material for: Prevalence of DSPN and Its Risk Factors Among Type 2 Diabetes Patients Attending Xuhang Community Health Service Center, in Shanghai, China
Source: Int J Endocrinol. 2026 Apr 17;2026:5704920. doi: 10.1155/ije/5704920 (PMC13088976; doi:10.1155/ije/5704920)
Supplement: Supplementary file 2 — Supporting Information 2 Supporting Information 2 contains screening tests for peripheral neuropathy, lower extremity vascular examination, and skin condition and toe abnormalities. [file IJE-2026-5704920-s003.docx]

**Supplementary material 2**

**2.1 5** **screening tests for peripheral neuropathy**

**Pressure Sensation:** Initially, apply the 10g monofilament to the patient's hand to acclimate them to the sensation of the nylon filament. Subsequently, instruct the patient to close their eyes to ensure they cannot observe the examiner's procedure. Apply the monofilament vertically to the base of the first metatarsal with sufficient force until it bends into a "C" shape, holding this position for 1–2 seconds. Avoid direct contact with ulcers, wound healing areas, scars, or necrotic tissues. Inquire whether the patient perceives pressure and its specific location. Conduct three tests using the monofilament, ensuring that one of these tests is a simulated test (without making contact with the patient's skin).

If two of the three tests are answered correctly, this confirms that the pressure sensation is normal; if both relevant responses are incorrect, this indicates an abnormality in pressure sensation.

**Vibration Sensation:** Prior to the procedure, place the tuning fork on the patient's hand to familiarize them with the sensation of vibration. Then, instruct the patient to close their eyes to ensure they cannot observe the examiner's actions. Position the vibrating tuning fork vertically at the bony prominence of the metatarsophalangeal joint of the great toe, asking the patient whether they perceive the vibration (regardless of whether they do or do not, they must report it).

If the vibration of the tuning fork can be perceived for a duration of 5 seconds or longer, the vibration sense is considered normal; if it is less than 5 seconds, the vibration sense is deemed abnormal.

**Pinprick Sensation:** Begin by lightly pricking the skin of the patient's upper arm or olecranon process as a control. Next, lightly prick the skin on the dorsum of the patient's great toe, inquiring whether they perceive a "sharp stabbing pain."

The ability to feel pain upon needle pricking is considered normal, while the inability to feel pain is regarded as abnormal.

**Thermal Sensation:** Conduct a control test by contacting the skin of the patient's upper arm or olecranon process with the detector. While the patient has their eyes closed, place the two ends of the detector (the cool metal end and the warm polyester end) at any point on the dorsal skin of the foot for 1–2 seconds of continuous pressing for detection. Avoid abnormal areas such as calluses, ulcers, scars, and necrotic tissues.

The ability to distinguish cold and warmth indicates normal thermal sensation, whereas an inability to perceive these stimuli suggests a thermal sensation abnormality.
**Ankle Reflex:** The patient lies supine, flexing one knee to approximately 90 degrees with the leg naturally abducted. The examiner uses their left hand to dorsiflex the foot by grasping the forefoot, and strikes the Achilles tendon with a reflex hammer held in their right hand.

If the reflex can be clearly elicited, it is determined that the ankle reflex is normal. If ankle joint flexion movement is minimal or absent despite increased force, the ankle reflex is deemed to be diminished or absent.

All examinations were performed bilaterally.

**2.2 Lower extremity vascular examination**

**Dorsalis pedis artery :** The dorsalis pedis artery is located at the midpoint of the line connecting the medial and lateral malleoli, between the extensor hallucis longus tendon and the extensor digitorum longus tendon (on the middle of the dorsum of the foot, between the first and second metatarsals). Bilateral symmetry of the pulsations can be assessed by applying equal pressure with the index, middle, and ring fingers of both hands.

Mild to moderate symmetrical bilateral pulsation is considered normal, whereas asymmetry, weakening, or disappearance of pulsation is deemed abnormal.

**Posterior tibial:** The posterior tibial artery can be palpated approximately 2.5 cm posterior to the medial malleolus (anterior to the Achilles tendon). Bilateral symmetry of the pulsations can be assessed by applying equal pressure with the index, middle, and ring fingers of both hands.

Mild to moderate symmetrical bilateral pulsation is considered normal, whereas asymmetry, weakening, or disappearance of pulsation is deemed abnormal.

**Popliteal artery:** The popliteal artery is located relatively deep and requires stabilizing the knee joint with both hands, placing the thumbs on either side of the patella, and applying pressure with the fingertips of the other four fingers toward the center of the popliteal fossa for palpation.

Mild to moderate symmetrical bilateral pulsation is considered normal, whereas asymmetry, weakening, or disappearance of pulsation is deemed abnormal.

**2.3** **Skin condition and toe abnormalities：**

**Dry skin:** The skin exhibits localized tightness; obvious desquamation is not visible to the naked eye, yet a slight roughness can be detected upon palpation.

**Chap:** The skin surface exhibits cracks of varying lengths, ranging from fine, superficial fissures akin to paper cuts to deeper fissures extending several millimeters. The edges of these cracks are irregular and rough, with a base that appears erythematous or may ooze blood. These lesions are frequently accompanied by dryness, desquamation, and hyperkeratosis of the surrounding skin.

**Furfur:** The skin surface exhibits white or grayish-white flaky or fragmented scales. Upon palpation, the skin feels rough, and additional scales can be easily dislodged through gentle scraping.

**Corn:** A round or oval-shaped, pale yellow hard nodule with well-defined margins and a smooth or slightly rough surface. A conical, translucent keratin plug is centrally located within the nodule, deeply embedded in the skin. Vertical pressure on the nodule elicits significant pain.

**Callus:** A localized keratotic plaque with a yellowish hue exhibits central thickening, peripheral thinning, and poorly defined margins. The surface is firm to the touch, while skin markings remain clearly visible. Local sweat secretion is diminished, leading to reduced tactile sensitivity. The lesion is asymptomatic, with no tenderness upon palpation.

**Malformation:** In this study, the term "malformation" is specifically used to denote toe deformities, encompassing various abnormalities in the shape, structure, or alignment of the toes. If any of the following 4 situations exist, then it can be determined that there is a toe deformity.

(1) Hammer toe: It is characterized by flexion contracture of the proximal interphalangeal joints of the toes, hyperextension of the metatarsophalangeal joints with occasional dislocation, and neutral or dorsiflexed positioning of the distal interphalangeal joints.

(2) Mallet toe: The primary feature is the flexion deformity of the distal interphalangeal joints (those nearest to the toe tips), which results in a "drooping" appearance of the toe tips. Meanwhile, the metatarsophalangeal and proximal interphalangeal joints typically remain in a neutral alignment, forming an "inverted V" shape when viewed from the side.

(3) Claw toe: It is characterized by dorsiflexion of the metatarsophalangeal joint, with both the distal and proximal interphalangeal joints in a flexed position.

(4) Hallux valgus: It is typically characterized by lateral deviation of the big toe at the first metatarsophalangeal joint, accompanied by the development of prominent bone spurs on the medial aspect of the joint.
