# Supplementary material for: Prevalence of DSPN and Its Risk Factors Among Type 2 Diabetes Patients Attending Xuhang Community Health Service Center, in Shanghai, China
Source: Int J Endocrinol. 2026 Apr 17;2026:5704920. doi: 10.1155/ije/5704920 (PMC13088976; doi:10.1155/ije/5704920)
Supplement: Supplementary file 3 — Supporting Information 3 Supporting Information 3 includes the R packages and commands used for statistical analysis and graphing. [file IJE-2026-5704920-s001.docx]

library(haven)

basic <- read_sav("/basic.sav")

library(tidyverse)

df <- basic

library(tableone)

CreateTableOne(data=df)

CreateTableOne(strata='dpn',data=df)

glm(dpn~age+duration+CVD+dry+callus+DPAP+PTAP+PP+LDL+Cr,data=df,family = binomial)

library(rms)

lrm(dpn ~ age + duration + CVD + dry + callus + DPAP + PTAP + PP + LDL + Cr,data=df,x=T,y=T) ->a

dd<-datadist(df)

options(datadist'dd')

options(datadist='dd')

nomo1<-nomogram(a,fun=plogis, funlabel = 'Risk of DSPN')

plot(nomo1,col.grid = c('tomato','darkcyan'))

library(readxl)

pos <- read_excel("pos.xlsx")

View(pos)

library(ggplot2)

View(pos)

order<-sort(pos$positive,index.return=TRUE,decreasing = FALSE)

View(order)

pos$item<-factor(pos$item,levels = pos$item[order$ix])

View(pos)

ggplot(pos,aes(positive,item))+geom_point(shape=21,fill='red')+geom_segment(aes(x=0, xend=positive, y=item, yend=item))

library(forestmodel)

forest_model(a)
